# Attachment Style Is Related to Quality of Life for Assistance Dog Owners

**DOI:** 10.3390/ijerph14060658

**Published:** 2017-06-19

**Authors:** Naomi White, Daniel Mills, Sophie Hall

**Affiliations:** School of Life Sciences, University of Lincoln, Lincoln LN6 7DL, UK; naomiwhite16@hotmail.com (N.W.); dmills@lincoln.ac.uk (D.M.)

**Keywords:** dog attachment, assistance dogs, quality of life, anxious attachment

## Abstract

Attachment styles have been shown to affect quality of life. Growing interest in the value of companion animals highlights that owning a dog can also affect quality of life, yet little research has explored the role of the attachment bond in affecting the relationship between dog ownership and quality of life. Given that the impact of dog ownership on quality of life may be greater for assistance dog owners than pet dog owners, we explored how anxious attachment and avoidance attachment styles to an assistance dog affected owner quality of life (*n* = 73). Regression analysis revealed that higher anxious attachment to the dog predicted enhanced quality of life. It is suggested that the unique, interdependent relationship between an individual and their assistance dog may mean that an anxious attachment style is not necessarily detrimental. Feelings that indicate attachment insecurity in other relationships may reflect more positive aspects of the assistance dog owner relationship, such as the level of support that the dog provides its owner.

## 1. Introduction

There is growing recognition that owning a pet can benefit individual well-being [1,2,3,4,5,6]. Although mechanisms underlying the “pet-effect” are unclear, one theory which has received considerable attention is that pets act as secure attachment figures, providing comfort and support in times of need [7,8,9]. Attachment styles are shown to be significant predictors of quality of life across a range of ailments and demographic factors [10,11,12,13,14]. One cohort of individuals who may, in particular, experience the benefits of attachment to a pet are those who own an assistance dog, since these individuals rely on their dog for support with essential daily tasks. However, research in this area is primarily focussed on the benefits that assistance dogs bring to their owners’ immediate physical daily needs, as opposed to broader quality of life.

The concept of an attachment figure was first applied to describe parent child relationships [15]. It was later apparent that elements of attachment are applicable to a range of social behaviours and cognitions [16], such as those occurring between romantic partners [17], patients and therapists [18], and in organisational settings [19]. There are four elements to a relationship which are essential criteria for the demonstration of an attachment bond: (a) proximity seeking and maintenance—preferring to stay close to an attachment figure, particularly during stressful times; (b) safe haven—turning to an attachment figure when distressed, to find comfort and support; (c) secure base—using an attachment figure as a base from which to explore an unfamiliar environment or unknown situation; (d) separation anxiety—resisting and becoming distressed when separated from an attachment figure [20].

Recent research suggests that the relationship between owner and dog can satisfy the criteria of an attachment bond [8,9,21,22,23,24,25]. Specifically, people enjoy feeling close to their pets and try to maintain this closeness [26,27,28]. Owners often consider their pets as safe havens [28,29], and act as a secure base, allowing them to explore and take risks more confidently [30]. Separation from a pet, such as its death, can cause many owners to grieve and mourn its loss [31,32].

Caregiving and support are common features of a parent child attachment bond and these are also features which can be identified in the relationship between an assistance dog and its owner [33]. The reciprocal caregiving occurring between an assistance dog and owner is a particularly strong feature of the relationship between these two, and thus it might be predicted that attachment and quality of life are closely linked in this case. A small study of 25 physical assistance dog owners has highlighted how the responsibility of care for the dog may increase the owner’s sense of purpose in life [33] and this can be expected to have positive consequences for their quality of life [34]. Some people may find that their ability to perform daily activities is reduced by their disability, which takes away an element of control in their life [35]. Assistance dogs can be trained to help with many of these daily activities, including opening doors, retrieving items, and operating switches [36] reducing the amount of human assistance required and increasing feelings of independence and control [37]. The independence that such an assistance dog can bring to an owner may also result in wider benefits associated with improved quality of life, including better social integration, increased social acknowledgement, occupational changes, increased positive affect, and boosted confidence and self-esteem [38,39,40]. Despite this, little research has directly explored the impact of assistance dogs on quality of life [41,42] and especially the potential influence of the attachment bond on these benefits.

Although there are clear links between attachment and quality of life in centred relationships [43,44,45,46], there is little research which has explored whether attachment to a dog is related to owner quality of life. Zilcha-Mano et al. [8] reported that in a sample of Israeli pet owners, higher anxious attachment to a pet was associated with lower psychological well-being and greater psychological distress, and arguments may be made for the causal direction of this association being either way (i.e., greater anxious attachment resulting in poorer quality of life or aspects of a poorer quality of life such as tendency to worry, resulting in a greater tendency towards anxious attachment). However, the assistance dog owner relationship is more managed, with both having to meet certain suitability criteria before a match is made, and there is an expectation that the dog will become an instrumental part of the owner’s daily life and functional capacity. It is therefore important to specifically understand the nature of any link between attachment to an assistance dog and the quality of life of its owner, in order to maximise the benefits of this partnership to both. Therefore, the aim of this study was to investigate whether anxious attachment (e.g., concern about pet’s love, loyalty or availability) or avoidant attachment (e.g., striving to maintain emotional distance from pet) to their assistance dog was related to their quality of life and if so, in what way.

## 2. Materials and Methods

### 2.1. Ethics, Consent and Permissions

All subjects gave their informed consent for inclusion before they participated in the study. The study was conducted in accordance with the Declaration of Helsinki, and complied with the British Psychological Society (BPS) Ethics Code of Conduct (code: COSREC-2015-06) [47]. The protocol was approved by the delegated authority of the College of Science Ethics Committee, University of Lincoln.

### 2.2. Design and Materials

A survey was designed comprising a demographic questionnaire (age group, gender, and length of ownership of the dog), the Pet Attachment Questionnaire [8] and the Quality of Life Scale [48].

The Pet Attachment Questionnaire (PAQ) [8]: This questionnaire has been designed to measure an individual’s attachment style to their pet. The 26-item self-report measure assesses avoidance (13 items: e.g., “I prefer not to be too close to my pet”) and anxious (13 items: e.g., “I need a lot of reassurance from my pet that it loves me”) dimensions of attachment insecurity. The scores on each subscale are summed, with higher scores indicating higher attachment insecurity (one item on the avoidance scale is reverse scored). The items relate to how a person feels about their relationship with their dog. Each item is rated on a scale of 1–7 (1 = strongly disagree; 7 = strongly agree). The questionnaire has reports of good internal and test-retest reliability [8].

The Quality of Life Scale (QoLS) [48]: This 16-item scale was used to measure an individual’s quality of life. On a 7-point scale (1 = terrible/highly dissatisfied; 7 = delighted/highly satisfied) individual rates how satisfied they feel about items, which relate to health, work, close friends, and independence; scores are summed together to give a single quality of life score. The scale has reports of good reliability and validity [48].

### 2.3. Participants

Participants were self-recruited from the database held by the United Kingdom (UK) charitable organisation Dogs for Good. From a possible 181 participants’, responses were obtained from 80 participants (response rate: 44%), 7 questionnaires were not included in analysis due to multiple (more than two) missing answers. Of the remaining 73 participants, 17 were male (24%) and 55 were female (76%), one participant did not state their gender. The most widely represented age group was 55–64 years (32.9%), followed by 45–55 years (30.1%), 35–55 years and over 65 years (both 6.4%), the least represented age groups were 25–34 years (2.7%) and 18–24 years (1.3%). The majority of respondents had owned their assistance dog for 2 years (18%), followed by 1 year (16.7%), 6 years and 8 years (both 11.1%), 7 years (9.7%), 3 and 4 years (both 8.3%), 9 years (6.9%), 5 years (5.6%), and 10 plus years (4.2%).

### 2.4. Procedure

The survey was sent via the postal system to all Dogs for Good’s Adult Assistance Dog partnerships (*n* = 181) by the charity, with a cover note explaining the nature of the study and request for help. Participants were provided with a pre-paid envelope and asked to return the completed surveys within the next few days to the University of Lincoln. To protect the participants from feeling pressured to take part in the survey no reminder messages were sent, and it was made clear that participation was entirely voluntary. Any responses returned after the closure date were not included in the analysis.

### 2.5. Data Analysis

Statistical analyses were performed using SPSS (IBM Corporation, Armonk, NY, USA), version 22. In total, 12 participants had two or one missing response on the PAQ (those with more than two were excluded); for these cases the “nearest neighbor” imputation technique was used, in which the missing score was imputed from the previous individual who had given the same score on the previous item. To assess the nature of the relationship between quality of life scores, pet attachment, gender, and the length of time dog an ordinal logistic regression analysis was performed. Significant *p* values were deemed as *p* < 0.05.

## 3. Results

Descriptive statistics for scores of pet attachment and quality of life are reported in Table 1, as a total group and separately for gender, age groups and length of time dog owned.

In order to understand the effects of gender and length of ownership, attachment avoidance, and attachment anxiety on quality of life in owners of assistance dogs, an ordinal logistic regression was run. A backward stepwise method was used, successively eliminating non-significant (*p* > 0.05) variables, in order to generate the minimum adequate model. Avoidant attachment (*p* = 0.61), length of ownership (*p* = 0.47) and gender (*p* = 0.17) were excluded from the final model. However, anxious attachment was a significant predictor of quality of life (*p* = 0.01; Table 2). The odds ratio indicates that as anxious attachment increased, quality of life also increased. Although the age group 35–44 years produced a statistically significant *p* value (*p* = 0.02), the large confidence intervals associated with this *p* value (95% lower confidence interval (CI): 1.91, upper CI: 2966.55) suggests a low level of precision of the odds ratio (75.35). Therefore, we do not report or discuss the effect of age further. The Pearson’s chi-squared goodness of fit test showed that the model fitted the data well (*p* = 0.94; Table 3).

## 4. Discussion

With the aim of assessing whether attachment to an assistance dog affects owner’s quality of life we collected data on pet attachment (avoidance attachment and anxious attachment to the assistance dog) and quality of life from assistance dog owners registered with the charity Dogs for Good. Our results showed that greater anxious attachment to an assistance dog predicted higher quality of life, suggesting that for assistance dog owner’s anxious attachment has a positive effect on perceived quality of life.

This is the first study, known to the authors, which explores whether attachment to an assistance dog affects owner’s quality of life. Our results are in direct contrast to those exploring this association in a sample of pet dog owners, with no known disabilities [8]. These contrasting results may reflect cultural differences between the two samples (UK vs. Israel), or the difference in sample size, with our study sampling less than half of that by Zilcha-Mano (Study 1, *n* = 302; Study 2, *n* = 212) [8]. Another possible explanation relates to differences in the nature of the dog owner relationship for pet owners and those with a trained assistance dog. For pet dog owners, anxious attachment may be a reflection of more pervasive worrying and fear that something bad may happen to their pet [8], or associated with problem behaviour, which should not be a feature of the assistance dog. Preoccupation with a need to provide protection and support may cause heightened levels of distress [45] and so result in a reduced quality of life. In contrast, the greater support networks associated with assistance dog ownership [36,37] may buffer these owners from such tendencies [49]. Another possible explanation is that the degree of anxious attachment shown by the assistance dog owners reflects the degree to which the assistance dog supports the individual to lead a fulfilling and independent life (i.e., greater quality of life). In this context, the anxious attachment is the inevitable cost of awareness of the benefits derived from an assistance dog, but may be framed in a more positive way so it does not negate the quality of life benefits of owning the dog. Future investigation which identifies whether or in what circumstances anxious attachment relates to psychological health, as opposed to general quality of life, may help to test elucidate this further.

Given that assistance dog owners rely—and, in some sense, have chosen to rely—on their dogs for support and physical assistance it is not surprising that scores on the avoidance attachment scale were lower than those on the anxious attachment scale. The mean scores were almost as low as they could be (lowest possible score is 16), indicating that avoidance attachment is not a feature of the attachment bond between the owner and dog, but this does not mean it is not important in other owner dog relationships.

This study represents an important first step for exploring how the relationship between owner and assistance dog affects well-being outcomes. However, as discussed, whilst this provides a general, global parameter of well-being, future research should consider using a range of specific psychological scales, such as those designed to measures depression, anxiety and distress. Additionally, although this study provides an initial investigation in the area, we recognise the sample size was relatively small, but this in part reflects the limited population of registered assistance dogs for those with physical disability in the UK, there being only one other major charity also providing dogs for this purpose. We acknowledge that this study used subjective self-report assessments of quality of life; this was largely because of their simplicity and perceived quality of life has been shown to be a strong predictor of mortality [50,51]. Nonetheless, our findings indicate it would be worth investing in more objective measures (e.g., use of health services) in future studies, in order to predict these and other effects of interest.

## 5. Conclusions

In conclusion, anxious attachment to an assistance dog, unlike a pet dog, is associated with greater perceived quality of life. This is probably due to different phenomenological features underlying the cause of anxious attachment in the two contexts, and we hypothesise that, within an assistance dog partnership the anxiety may be incidental and perhaps the inevitable cost associated with the service provided by the dog within the context of a reciprocally caring partnership. Thus, the relationship between attachment insecurity and quality of life appears to be highly dependent on the context and on the individual.

## Figures and Tables

**Table 1:** Descriptive statistics (means ± standard error means) for scores of quality of life, avoidance pet attachment and anxious pet attachment.

Caption	Quality of Life Scale (QoLS) ^1^	Avoidance Pet Attachment ^2^	Anxious Pet Attachment ^2^
Total sample (*n =* 73)	77.79 ± 1.76	16.82 ± 0.60	37.97 ± 1.36
Males (*n =* 17)	76.88 ± 4.19	18.76 ± 1.68	35.35 ± 2.55
Females (*n =* 55)	77.67 ± 1.93	16.29 ± 0.59	38.92 ± 1.61
18–24 years (*n =* 1)	97.00 ± 0.00	13.00 ± 0.00	42.00 ± 0.00
25–34 years (*n =* 2)	89.00 ± 4.00	18.00 ± 3.00	42.50 ± 0.00
35–44 years (*n =* 12)	69.50 ± 3.22	17.00 ± 1.23	31.00 ± 2.43
45–54 years (*n =* 22)	77.22 ± 3.26	17.40 ± 1.24	36.86 ± 2.13
55–64 years (*n =* 24)	75.91 ± 3.07	15.83 ± 0.84	40.25 ± 2.84
≥65 years (*n =* 12)	87.41 ± 4.05	17.66 ± 2.04	41.33 ± 2.85
Owned dog for 1 year (*n =* 12)	71.66 ± 5.18	17.00 ± 1.57	38.33 ± 2.98
Owned dog for 2 years (*n =* 13)	85.13 ± 3.47	16.15 ± 1.25	33.61 ± 3.88
Owned dog for 3 years (*n =* 6)	78.00 ± 6.41	16.16 ± 2.00	40.00 ± 4.06
Owned dog for 4 years (*n =* 6)	90.83 ± 5.96	14.66 ± 1.47	28.16 ± 1.47
Owned dog for 5 years (*n =* 4)	71.25 ± 5.17	15.00 ± 1.35	43.50 ± 6.18
Owned dog for 6 years (*n =* 8)	74.75 ± 5.47	18.12 ± 2.60	43.75 ± 2.56
Owned dog for 7 years (*n =* 7)	79.85 ± 4.46	17.14 ± 1.79	46.14 ± 3.44
Owed dog for 8 years (*n =* 8)	67.00 ± 4.98	16.25 ± 1.09	42.37 ± 3.61
Owned dog for 9 years (*n =* 5)	87.80 ± 3.27	17.20 ± 1.62	25.60 ± 1.86
Owned dog for ≥10 years (*n =* 3)	71.66 ± 3.92	18.00 ± 5.00	35.66 ± 7.85

^1^ Quality of Life Scale (QoLS; [48]): Maximum total score = 112. ^2^ Pet Attachment Questionnaire ([8]): Maximum total score on each subscale = 91.

**Table 2:** Significant predictors of quality of life scores using a backwards stepwise regression analysis.

Predictor	B	β	*z*	*p*	Odds Ratio	Lower CI *	Upper CI
Constant	−0.01	2.09	−0.00	0.99			
Anxious attachment	0.05	0.02	2.73	0.01	1.05	1.01	1.09

* Confidence interval: 95%; B: Unstandardized Beta Coefficient; β: Standardized Beta Coefficient; *z*: Z-statistic; *p*: Probability (*p* < 0.05).

**Table 3:** Model evaluation and goodness of fit statistics.

Test	*x*^2^	df	*p*
Log-Likelihood	−254.02	6	0.002
Pearson’s chi-squared	2212.33	2316	0.938

*x*^2^: Chi-square statistic; df: degrees of freedom; *p*: Probability (*p <* 0.05).
